# Surgical treatment of Chiari I malformation complicated with syringomyelia

**DOI:** 10.3892/etm.2012.784

**Published:** 2012-10-31

**Authors:** CHANGSHUN BAO, FUBING YANG, LIANG LIU, BING WANG, DINGJUN LI, YINGJIANG GU, SHULING ZHANG, LIGANG CHEN

**Affiliations:** Department of Neurosurgery, The Affiliated Hospital of Luzhou Medical College, Luzhou, Sichuan 646000, P.R. China

**Keywords:** Chiari malformation, syringomyelia, posterior fossa decompression, duraplasty

## Abstract

The aim of this study was to evaluate the curative effects of various surgical procedures on Chiari I malformation (CMI) complicated with syringomyelia. A total of 185 patients with CMI complicated with syringomyelia who received treatment between January 1997 and December 2011 were recruited. All patients underwent posterior fossa decompression in which the lamina of the first cervical vertebra was removed, with the removal of the second or third depending on the severity of the cerebellar tonsil herniation. Of the patients, 76 underwent large-bone-window decompression and duraplasty, while 109 underwent small-bone-window decompression, displaced cerebellar tonsil resection and duraplasty. The curative effects of the different surgical procedures were analyzed retrospectively. Clinical symptoms were eliminated or improved in 156 patients (84.3%) by the time of discharge from hospital. A total of 148 patients were evaluated using magnetic resonance imaging (MRI) which revealed that the cisterna magna was reconstructed in 92 patients and spinal syrinx was reduced in 75. Follow-up was performed on 147 patients (79.5%) for between 3 months and 12 years. During the follow-up, symptoms were eliminated or improved in 110 patients (74.8%), not improved in 26 (17.7%) and deteriorated in 11 (7.5%). MRI was performed on 95 patients during follow-up examinations and the cisterna magna was reconstructed in 87 patients and spinal syrinx was reduced in 79. Small-bone-window decompression plus duraplasty is an effective surgical procedure for treating CMI complicated with syringomyelia and intraoperative cerebellar tonsillectomy significantly aids patient recovery.

## Introduction

Chiari malformation (CM), also known as Arnold-Chiari malformation, is a congenital developmental malformation characterized by a downward displacement of the cerebellar tonsils into the spinal canal due to the reduced capacity of the posterior cranial fossa. CM may be complicated by a variety of other malformations, including platybasia, basilar invagination and occipitalization, although syringomyelia (SM) is the most commonly observed.

In 1883, Cleland first described brainstem and cerebellar displacements and SM (1). Chiari made successive attempts to classify these malformations into various types according to the severity of the downward displacement of the neural axis in the skull-vertebra transitional area and types I–IV were defined: i) type I, displacement of the cerebellar tonsils and the medial portions of the inferior lobes of the cerebellum which follow the bulb inside the cervical canal; ii) type II, displacement of the lower portions of the cerebellum, pons, medulla and part of the elongated IV ventricle inside the cervical canal; iii) type III, significant portions of the cerebellum and brainstem are dislocated caudally and the orifices of the IV ventricle open into the cervical canal, reshaping the cervical hydrocephalus through spina bifida of the first three cervical vertebrae; and iv) type IV, hypoplasia of the cerebellum without a caudal displacement of the brainstem (2,3). Later, in 1894, Arnold, a German pathologist, added further detail to the descriptions of these malformations (4). Schwalbe and Gredig suggested the term ‘Arnold-Chiari malformation’ to refer to the condition in 1907 (5), whereas Sarnat and Williams named it Chiari malformation (CM) or cerebellar tonsil downward displacement malformation in terms of the primary clinical manifestation of the condition (6,7). Further studies have since been dedicated to evaluating the occipitocervical malformations (8–10).

CM manifests as a variety of clinical symptoms, principally including spinal canal impairment, a dissociated sensory disorder of the limbs and body and muscular atrophy (particularly of the upper limbs); nerve root irritation, causing painful or burning sensations in the neck, shoulders, back or upper limbs; posterior group cranial neural and cerebellar disorders, causing instability of gait, nystagmus, dysphagia and hoarseness; pyramidal tract impairment, causing hypermyotonia, tendon hyperreflexia and loss of muscle strength; and intracranial hypertension, causing headaches, emesis and papilledema. At present, CM diagnosis is mainly based on a combination of clinical manifestations and magnetic resonance imaging (MRI) of the occipitocervical area. The radiographic criteria for diagnosing CM include cerebellar tonsillar herniation into the spinal canal (a downward herniation of the cerebellar tonsils >5 mm below the foramen magnum), decreased posterior fossa capacity, cisterna magna shrinkage or disappearance, and compression against and malformations of the cervical cord and IV ventricle or displacements toward the spinal canal. For patients with confirmed CM, surgery is the only effective therapeutic measure, where decompression of the suboccipital region is performed in order to re-form the cisterna magnum. However, since certain patients exhibit no neurological improvement, surgical treatment remains controversial with regard to the size of the bone window for decompression, whether the dura mater should be opened, how the herniated tonsils should be treated and whether syrinx drainage is required.

The present study was intended to resolve these issues concerning the surgical treatment of CMI based on clinical experience.

## Patients and methods

### General data

A total of 185 patients with confirmed CMI (atlantoaxial dislocation and occipitocervical instability were excluded) were enrolled in the present study. Of the patients, 82 were male and 103 were female. The duration of the disease ranged between 7 days and 12 years with an average of 3.7 years. The patients’ ages ranged between 15 and 68 years with an average of 43.5 years. CMI occurred in 21 patients <21 years old, 118 (63.8%) between 25 and 45 and 46 of >45.

The study was conducted in accordance with the Declaration of Helsinki and approved by the Ethics Committee of the Affiliated Hospital of Luzhou Medical College (Luzhou, China). Written informed consent was obtained from all participants.

### Clinical manifestations

Clinical manifestations were as follows: i) spinal canal impairment symptoms: 132 patients exhibited dissociated sensory disorders of the limbs and body and 61 exhibited muscle atrophy in the hands or upper limbs; ii) nerve root irritation symptoms: 74 patients had painful and burning sensations in the neck, shoulders, back or upper limbs; iii) posterior group cranial neural and cerebellar disorders: instability of gait, nystagmus, and dysphagia and hoarseness were observed in 36, 24 and 16 patients, respectively; iv) pyramidal tract impairment symptoms: 68 patients were diagnosed with hypermyotonia, tendon hyperreflexia and loss of muscle strength; and v) increased intracranial pressure signs and symptoms: 16 patients were identified as having headaches, emesis and papilledema.

### MRI

Occipitocervical MRI revealed that the downward herniations of the cerebellar tonsils in the patients ranged from 3 to 19 mm with an average of 9.35 mm. A total of 146 patients had a herniation >5 mm below the lower border of the foramen magnum and 39 had a herniation 3–5 mm below. A spinal syrinx confined to the cervical region was observed in 46 patients, while 139 had a cervical and thoracic syrinx. The ratio between the diameter of the spinal syrinx and that of the spinal cord was <0.35 for 132 patients and 53 had a ratio of >0.35. All patients exhibited a marked decrease in the size of or even the disappearance of the cisterna magna. The complications observed were as follows: 51 patients with basilar impression, 38 with platybasia, 32 with occipitalization, 67 with scoliosis, 16 with neuropathic arthropathy and 17 with hydrocephalus.

### Surgical procedures

All patients underwent surgery in a prone position with the head fixed using a head rest and the neck slightly forward, following general anesthesia with tracheal intubation.

Large-bone-window posterior fossa decompression plus duraplasty was performed on 76 patients. A posterior median incision was made between 1 cm below the external occipital protuberance and the spinous process of the third cervical vertebra (C_3_) and the squamous part of the occipital bone was removed up to the posterior border of the foramen magnum. The processes and laminae of the C_1–3_ were excised depending on the severity of the cerebellar tonsil herniation. A 2.5-cm-wide section of the posterior border of the foramen magnum and 2-cm-wide section of the posterior arches of the C_1–3_ were removed. The thickened occipital fascia was excized under a microscope, the dura mater was Y-sheared and artificial dura mater or muscular fascia was then used to expand and repair the dura mater. The dura mater was suspended on the border of the bone window and shaped afterwards.

Small-bone-window posterior fossa decompression, cerebellar tonsillectomy and duraplasty were performed on 109 patients. The same surgical approach was adopted but with removal of a 3x3 cm section of the occipital bone. The dura mater was then Y-sheared and the adhesions of the arachnoid to the dura mater and tonsils and of the tonsils to the brainstem were separated under a microscope. Cerebellar tonsil electric coagulation was performed under such conditions that noticeable repositioning did not occur and the cerebellar tonsils up to 5–10 mm above the level of the foramen magnum were excised subspially to relieve compression against the medulla oblongata and cervical cord (attention was also paid to the relief of the compression of the outer sides of the cerebellar tonsils against the nerve roots). The median and lateral apertures of the IV ventricle were detected and possible adhesions were sufficiently released to guarantee unobstructed IV ventricular cerebrospinal fluid (CSF) circulation. The dura mater was repaired with artificial dura mater and then suspended on the border of the bone window and shaped into the cisterna magna by suturing the occipital muscle tissues. An external drainage tube was held outside the dura mater and the incision was sewn up.

Patients turned their bodies over axially following the surgery. The patients received neck fixation for 2–3 weeks.

## Results

### Short-term curative effects

The curative effects were evaluated after the treatment as well as at the time of hospital discharge. The lengths of the patients’ hospital stays ranged between 10 and 21 days with an average of 14 days. Symptoms were eliminated or improved in 156 patients (84.3%) and not improved in 29 (15.7%). No symptom deterioration or mortality occurred. Incisional hydrops were observed in 8 patients but were healed following drainage and dressing changes of the wound and no CSF leakage occurred. A total of 148 patients received MRI within two weeks after operation. The results revealed that 92 patients (62.2%) had a reconstructed cisterna magna and 75 (50.7%) had a reduced spinal syrinx.

### Long-term curative effects

**Figure 1:**
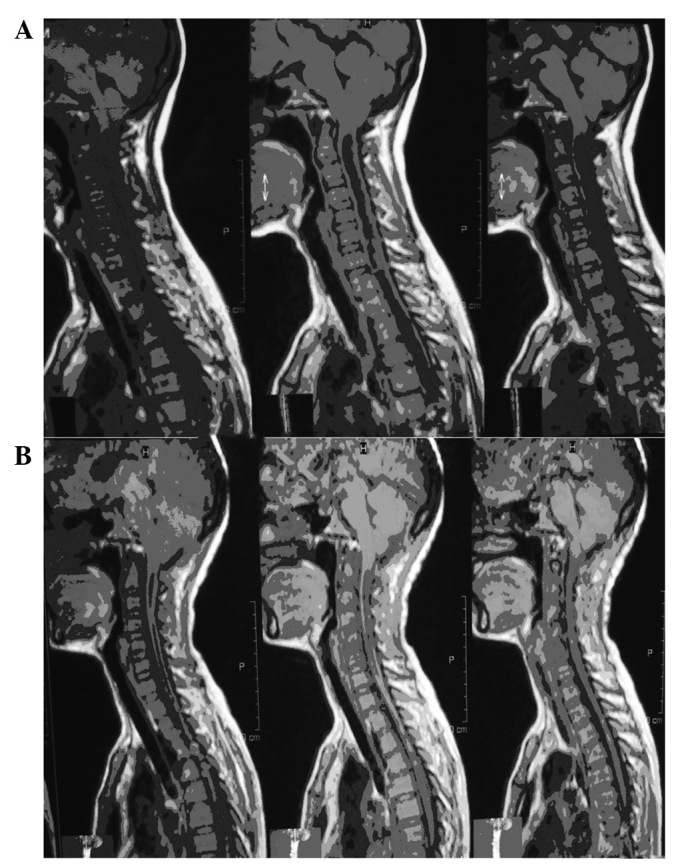
Preoperative and postoperative magnetic resonance images of patients with CMI complicated with SM. (A) Cerebellar tonsils have dropped below the foramen magnum to the atlantal level with severe SM prior to treatment. (B) Herniated cerebellar tonsils have moved upward to above the foramen magnum, the cisterna magna has effectively formed and the spinal syrinx has essentially disappeared 5 months after surgery. CMI, Chiari I malformation; SM, syringomyelia.

A total of 147 patients were followed up for 3 months to 12 years, with an average of 3.2 years and 38 patients (20.5%) were lost to follow-up. The symptoms were eliminated or improved in 110 patients (74.8%), not improved in 26 (17.7%) and aggravated in 11 (7.5%). MRI was performed for 95 patients and revealed that the cisterna magna was reconstructed in 87 patients and spinal syrinx was reduced in 79 (Fig. 1). Long-term complications, including headaches, fever (relieved after symptomatic treatment) and CSF leakage (healed after drainage, wound suturing and enhanced anti-inflammatory treatment) were exhibited by 8 of the patients. The 11 patients with aggravated symptoms primarily exhibited aggravation following symptom relief. MRI revealed that 8 of these patients still had a noticeable spinal syrinx with a ratio between its diameter and that of the spinal cord of >0.35. The patients underwent syringo-subarachnoidal (SS) shunting. The remaining 3 patients received further physical and symptomatic treatment. Symptoms were reduced somewhat in the majority of the patients.

## Discussion

A popular hypothesis concerning the pathogenesis of CMI is that the hindbrain tissues are dislocated into the spinal canal after birth due to an overcrowded posterior cranial fossa which is caused by the retarded development of the occipital bone during the embryonic period (11). However, the pathogenesis of SM remains controversial. The 3 main theories which attempt to explain the formation of SM are Gardner’s hydrokinetics, Williams’ intracranial and intraspinal pressure separation and Oldfield’s CSF and spinal substance penetration, none of which is superior to any other (12). Partial obstruction in the foramen magnum area blocks the normal circulation of CSF which is a major factor in the development and progression of SM (13). Morphological changes in the subarachnoid space are important in the development and progression of SM in that CM patients usually have increased atlanto-occipital fascia thickness and narrowed or even obstructed cisterna magnae, in addition to sclerotic structural abnormalities. The longer the duration of CM and the more severe the condition, the narrower the subarachnoid space (14). The false membrane at the orifice of the spinal canal is one of the causes of intraspinal canal fluid accumulation and the formation of a syrinx (8).

A reduced posterior fossa capacity and a narrowed occipitocervical subarachnoid space are key factors in the development of CMI complicated with SM. CMI is congenital, whereas SM is acquired. When the obstruction of the subarachnoid space reaches a certain extent, SM may occur. At present, the main treatment of CMI complicated with SM is surgery. However, surgical treatment only stops or retards the disease’s progression rather than curing the damage caused to the spinal cord. Therefore, patients exhibiting symptoms should be diagnosed and treated as early as possible. In the surgical treatment of CMI complicated with SM, posterior fossa decompression and SS shunting are two commonly-adopted procedures. With the establishment and development of the Oldfield theory, numerous studies have indicated that posterior fossa decompression is the preferred procedure. In this procedure, the key points include expanding the capacity of the posterior cranial fossa, reconstructing the absent cisterna magna and allowing the obstructed CSF to circulate through in order to make the syrinx disappear and improve the symptoms (Fig. 1) (15–17). By contrast, since SS shunting cannot aid the return of CSF circulation to normal and may also increase the risk of spinal cord injury and infections, despite a certain long-term curative effect, it is no longer recommended (15–18). The results of the present study support this since the follow-ups demonstrated that 79 out of 95 patients (83.2%) who did not undergo SS shunting exhibited a reduced spinal syrinx.

Previously, posterior fossa decompression alone was considered to be able to provide an efficient curative effect for CMI complicated with SM. However, although posterior fossa decompression alone expands the capacity of the posterior fossa, it fails to correct CSF pressure separation and reinstate normal CSF circulation. Therefore, posterior fossa decompression alone has essentially been excluded from clinical practice for treating CMI complicated with SM. Instead, a combination of posterior fossa decompression and duraplasty is widely used. The combined procedure efficiently solves the problem of a narrowed posterior fossa capacity by removing C_1–2_ and reshaping the dura mater through an occipital bone window, eliminating the compression in the foramen magnum area and improving the associated clinical symptoms. Numerous follow-up studies have demonstrated that posterior fossa decompression combined with duraplasty greatly improves the clinical symptoms of CMI with SM and reduces the spinal syrinx (16–18). Nevertheless, the size of the bone window for decompression is debated. An expanded bone window is crucial for treating CMI with SM (19). A small bone window for posterior fossa decompression is capable of achieving an effect as good as a large one and also reduces the incidence of postoperative complications. The present study revealed a higher incidence of complications in the patients receiving large-bone-window decompression and duraplasty. Early postoperative complications were primarily manifested as fevers, headaches, CSF leakage, pseudocyst and spinal arachnoiditis, whereas long-term postoperative complications mainly included diplopia, tinnitus, dizziness and limited neck activity. Expanded posterior fossa decompression and Y-shaped shearing and expanded repair to the dura mater possibly cause symptoms such as CSF leakage, subcutaneous hydrops and fevers. Additionally, the wide dissection of the occipital muscle, excessive removal of the squamous part of the occipital bone and the resulting backward displacements of the cerebellum, midbrain and medulla oblongata, which cannot gain support from the expansively-repaired dura mater, cause pseudocephalocele. This further pulls on the abducent, acoustic and trigeminal nerves, leading to a succession of long-term complications (20). In the present study, 109 patients underwent small-bone-window decompression and duraplasty. This procedure not only removes the compression of the occipitocervical bones and thickened fascia against the cerebellobulbaris but provides support for the cerebellum and midbrain to reduce the incidence of neuroses caused by their excessive drooping and dragging. Furthermore, this procedure requires less intraoperative muscular dissection which aids the recovery of the neck and nape (9).

In CMI, the cerebellar tonsils are the contents of the syrinx as well as the main cause of the compression against the medulla oblongata which further leads to SM and causes the associated clinical symptoms. Although the necessity of opening the arachnoid space to remove the herniated cerebellar tonsils in the treatment of CMI is debatable (17,21), the removal approach is preferred and is considered to achieve a more positive curative effect. The herniated cerebellar tonsils should be excised subpially, although attention should also be paid to the median and lateral apertures of the IV ventricle and adhesions should be sufficiently released to guarantee the smooth circulation of CSF. For patients with nerve root irritation symptoms, the compression of the cerebellar tonsils against the nerve roots should be relieved (22). In the present study, the recovery following surgery of the patients receiving herniated cerebellar tonsil exsection was superior to that of the patients who did not receive such a treatment. The patients who underwent cerebellar tonsil excision exhibited noticeably shortened recovery times for nerve root irritation and ataxia and superior muscle strength recovery. This result indicates that attempts may be made to remove the herniated cerebellar tonsils to aid patient recovery, on the assumption of adequate microsurgical experience.

Although satisfactory surgical effects were achieved in the majority of patients in the present study following treatment, certain patients did not exhibit significant improvement of symptoms, particularly spinal canal impairment. This suggests that surgical treatment only stops or retards rather than radically eliminates the progression of spinal impairment. Therefore, patients with symptoms should be diagnosed and surgically treated as early as possible (22).

In conclusion, CMI complicated with SM is a complex condition characterized by a variety of manifestations. At present, there is no agreement with regard to the surgical procedure for this condition and the curative effects of various procedures differ. With the development of studies of CMI complicated with SM, perfection of the surgical procedures and increased knowledge concerning the condition among medical practitioners, significant improvements in curative effects may be achieved.
